# HIV-1 integrase SUMOylation and viral replication

**DOI:** 10.1186/1742-4690-8-S2-O4

**Published:** 2011-10-03

**Authors:** Alessia Zamborlini, Audrey Coiffic, Guillaume Beauclair, Olivier Delelis, Joris Paris, Fabien Magne, Yashuiro Koh, Marie-Lou Giron, Joelle Tobaly-Tapiero, Stephane Emiliani, Alan Engelman, Hugues de The, Ali Saib

**Affiliations:** 1 CNRS UMR7212, INSERM U944, Université Paris7 Diderot, Institut Universitaire d'Hématologie, Paris, 75010, France; 2 Conservatoire National des Arts et Métiers, Department CASER, Paris, 75003, France; 3 LBPA, CNRS UMR8113 , Ecole Normale Supérieure, Cachin, 94235, France; 4 Dana-Farber Cancer Institute, Department of Cancer Immunology & AIDS, Boston, MA, 02115, USA; 5 INSERM U1016, CNRS JMR8104, Université Paris Descartes, Cochin Institut, Paris, 75014, France

## Background

HIV-1 integrase catalyzes the integration of the reverse transcribed viral cDNA into the cellular genome, a key event of retroviral replication that is targeted by novel anti-HIV therapeutic agents. Numerous studies have contributed to understand of the molecular basis of integrase catalytic functions. Besides integration, HIV-1 integrase participates in other steps of viral replication such as reverse transcription and/or uncoating, PIC nuclear import and virion morphology. However, the underlying mechanisms are still not fully elucidated. Cellular and viral factors assist integrase in performing its multiple activities. Moreover, post-translational modifications (i.e. acetylation, ubiquitination and phosphorylation), which represent a common, rapid and generally reversible mechanism for fine tuning of protein activities, have also been shown to regulate integrase functions.

## Materials and methods and results

By in vitro SUMOylation assay and purification on Ni-NTA beads in denaturing conditions, we show that HIV-1 integrase is covalently modified by the three SUMO paralogues. By mutating SUMO-acceptor lysine residues within phylogenetically conserved SUMOylation consensus motifs identified *in silico*, we demonstrate that they represent major sites of SUMO conjugation. We introduced the same changes in the integrase sequence of a plasmid encoding the viral genome, which was used to generate mutant viral particles, and we compare their infectivity to that of WT HIV-1. We find that viruses harboring SUMOylation-defective integrase mutants are less infectious that the WT counterpart. Real-time PCR analysis of viral cDNA synthesis reveals that cells infected with mutant viruses display similar content of traverse transcripts, but a lower number of integrated proviruses, than WT HIV-1-infected cells. This integration defect at the integration step is not due to impairment of integrase binding to LEDGF/p7S, a key chromatin-tethering factor of HIV-1 pre-integration complex. Indeed, both WT and SUMOylation-defective integrase proteins coprecipitated with similar efficiency with LEDGF/p75. Finally, we establish that SUMOylation-defective integrase mutants retained WT catalytic activity as determined by Vpr-fusion protein complementation assay.

## Conclusions

Here, we show that HIV-1 IN is modified by SUMO proteins and that phylogenetically conserved SUMOylation consensus motifs represent major SUMO-acceptor sites. Viruses harboring SUMOylation-site IN mutants displayed a replication defect that was mapped during the early stages of infection, before integration but after reverse transcription. Since SUMOylation-defective IN mutants retained WT catalytic activity as well as LEDGF-binding, we hypothesize that SUMOylation might regulate the affinity of IN for co-factors, contributing to efficient HIV-1 replication.

